# A Novel Closed-Loop Structure for Drag-Free Control Systems with ESKF and LQR

**DOI:** 10.3390/s23156766

**Published:** 2023-07-28

**Authors:** Xiaorong Ye, Junxiang Lian, Guoying Zhao, Dexuan Zhang

**Affiliations:** 1 TianQin Research Center for Gravitational Physics, Sun Yat-sen University, Zhuhai 519082, China; 2 School of Physics and Astronomy, Sun Yat-sen University, Zhuhai 519082, China

**Keywords:** extended state Kalman filter, linear quadratic regulator, drag-free control

## Abstract

Space-borne gravitational wave detection satellite confronts many uncertain perturbations, such as solar pressure, dilute atmospheric drag, etc. To realize an ultra-static and ultra-stable inertial benchmark achieved by a test-mass (TM) being free to move inside a spacecraft (S/C), the drag-free control system of S/C requires super high steady-state accuracies and dynamic performances. The Active Disturbance Rejection Control (ADRC) technique has a certain capability in solving problems with common perturbations, while there is still room for optimization in dealing with the complicated drag-free control problem. When faced with complex noises, the steady-state accuracy of the traditional control method is not good enough and the convergence speed of regulating process is not fast enough. In this paper, the optimized Active Disturbance Rejection Control technique is applied. With the extended state Kalman filter (ESKF) estimating the states and disturbances in real time, a novel closed-loop control structure is designed by combining the linear quadratic regulator (LQR) and ESKF, which can satisfy the design targets competently. The comparative analysis and simulation results show that the LQR controller designed in this paper has a faster response and a higher accuracy compared with the traditional nonlinear state error feedback (NSEF), which uses a deformation of weighting components of classical PID. The new drag-free control structure proposed in the paper can be used in future gravitational wave detection satellites.

## 1. Introduction

Gravitational wave astronomy provides a new tool to explore black holes, dark matter, the early universe, and the evolution of the universe. To detect gravitational waves in space, a strategy involves deploying multiple satellites in mega-satellite formations to measure tiny changes in the relative distances between satellites when the gravitational wave goes through. However, the challenge lies in the weak characteristics (to the order of $10^{-21}$) in the changes caused by gravitational waves, which can be easily influenced by extraneous perturbations and noise. To address this issue, the typical approach is to employ drag-free satellites: an inner test mass is shielded and free-falls along the geodesic of spacetime, and the outer satellite counteracts non-conservative forces and tracks the test mass in a sensitive axis. This setup creates an ultra-static and ultra-stable platform, with the test mass serving as an inertial reference for the measurement of relative distances in space. Using this approach, it is possible to obtain accurate and reliable measurements of distance variation. And the variation represents the effect of gravitational waves.

In the drag-free control loop, there are many factors that can impact the effect of the controller, such as external environmental disturbances, sensor measurement noise, process noise, and other inevitable disturbances and noises. Models of these disturbances and noises are hard to be built precisely, which makes it difficult to determine the appropriate models for compensation. To address this challenge, the Extended State Observer (ESO) method has emerged as an effective solution for modeling, estimating, and identifying disturbances in the drag-free control loop. Using the ESO method, it is possible to obtain estimates of the disturbances, which can help the design of the controller. This method has significant implications for the development of advanced control systems for drag-free satellites, thereby improving performance and reliability.

The Active Disturbance Rejection Control (ADRC) technique, proposed by Han [1,2], combines the “anti-disturbance” and “model independence” of PID control with the idea of the state observer. The Extended State Observer (ESO) is the core of ADRC, providing a way to estimate and compensate for disturbances and uncertainties. Huang [3,4] demonstrated the design method and proof of convergence for nonlinear ESO of second- and third-order systems, showing that it can achieve fast convergence without oscillation, even in the presence of model uncertainty and disturbances. However, the complexity of nonlinear ESO increases with the growth of the number of parameters, making tuning more challenging. Despite this drawback, the effectiveness of nonlinear ESO in mitigating disturbances and uncertainties makes it a promising technique for advanced control systems in various applications. Gao [5] proposed a parameter design method for linearized ESOs based on bandwidth, which effectively reduces the design threshold and improves the convenience of the application. Yang [6] analyzed the observation error of the ESO for different forms of disturbances and concludes that when the disturbance is bounded or its derivative is bounded, ESO can effectively estimate it, and the observation error is bounded. In addition, Jin [7], Chen [8], and Gan [9] analyzed the stability of the ESO using different methods, and Shao [10] analyzed high-order ESOs by adding higher-order derivative of disturbance as the extended state. Although increasing the extended order can effectively reduce the estimation error of each state, increasing the order and bandwidth simultaneously also affects the high-frequency noise suppression effect. Therefore, a trade-off between the expansion order and bandwidth is needed to balance the estimation accuracy and the high-frequency noise suppression effect. The Extended State Kalman filter (ESKF) proposed by Xue [11] combines the advantages of both extended state observer and Kalman filter to filter the noise and estimate the system state and disturbance in dealing with nonlinear systems with strong nonlinearity, large initial estimation error, and severe noise. The Extended State Kalman filter provides a potential solution to the problem of disturbance identification for the drag-free control of gravitational wave detection satellites, when the conventional filtering methods are not sufficient to estimate the disturbance.

The Linear Quadratic Regulator (LQR) is a widely used engineering tool in the aerospace industry due to its ability to achieve optimal control under specific performance requirements. Its simplicity in design has made it a popular choice for various applications, such as quadrotors [12,13,14,15,16], hypersonic vehicles [17,18], airborne remote gimbal [19], and satellite formation problems [20,21] etc.

In the field of control engineering, the Linear Quadratic Regulator (LQR) is widely used for linear problems. To ensure the effectiveness of the controller, an accurate linear model must be established or a nonlinear model should be linearized prior to the LQR design. In cases where low control performance requirements are sufficient, typically disturbances and noise are not handled directly but are instead compensated for through control. In order to improve the robustness and disturbance resistance of the traditional LQR controller, Lu [12] introduced the Extended State Observer (ESO) to estimate random low-frequency disturbances and the estimation of ESO is used by LQR. Attitude control of spacecraft with low precision requirements, disturbances, and noise are often not preprocessed and are only compensated for through LQR controllers. For systems with higher performance requirements, such as the six-degree-of-freedom attitude control system described in Ref. [17], a combination of ESO and LQR is used to achieve higher control accuracy and stronger disturbance resistance compared to using LQR control alone. In Lin’s research [19], a standard nonlinear ESO was employed in combination with LQR to estimate and compensate for multi-source perturbations, ultimately improving the control of LQR for uncertain systems. While these studies successfully combined an extended state observer with LQR control and achieved some improvement, they focused solely on the estimation and compensation of perturbations without considering the suppression of noise.

The accuracy required for drag-free and attitude control in gravitational wave detection is crucial, so the impact of noise must be considered. Previous methods are insufficient in dealing with the noise affecting gravitational wave detection satellites, and cannot estimate the perturbations effectively. Those methods also suffer from longer setting times. To design a successful control system, it is necessary to develop effective strategies to reduce the noise impact on control performance. In this study, we propose a novel approach that combines ESKF with LQR control. We use the state and disturbance estimated by ESKF as the input information for the controller, ensuring optimized control. Our analysis and simulations show that this new approach outperforms traditional solutions. It effectively shortens the adjustment time, reduces the number of oscillations, compensates for disturbances, and suppresses noise, ultimately achieving the desired design specifications.

The paper is organized as follows: in Section 2, a dynamic model of a single test mass drag-free satellite is established. In Section 3 and Section 4, the design process and calculation methods of the ESKF and LQR are presented, respectively. In Section 5, the performance of the system using the LQR controller and NSEF controller is compared through numerical simulations, indicating that the overall performance of the LQR controller is superior to that of the NSEF controller when using ESKF as the estimation method. The conclusion is given in Section 6.

## 2. Dynamics Modeling

**Figure 1:**
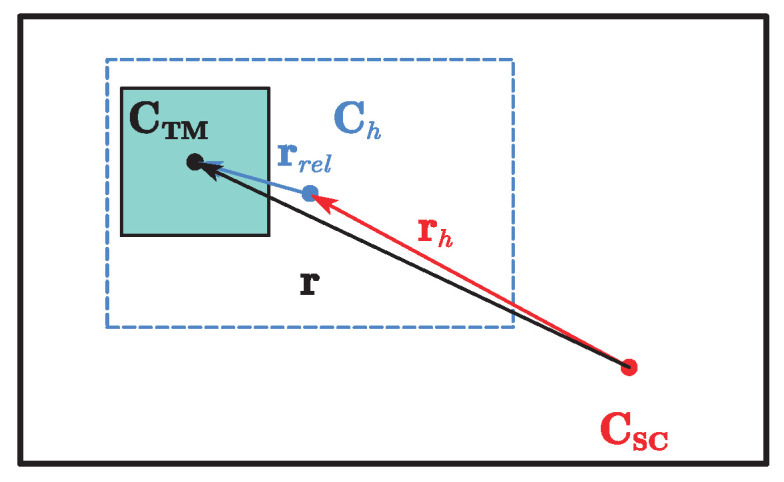
Relative positions of CoM of satellite, CoM of test mass, and center of cavity.

**Figure 2:**
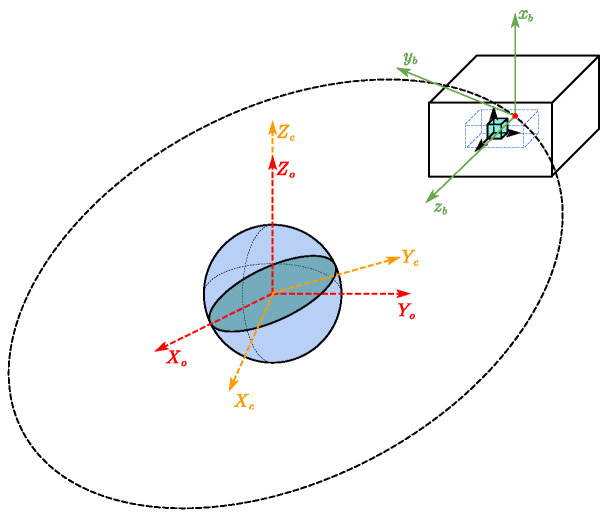
Coordinate system diagram.

This paper takes a single test mass, a drag-free satellite in geocentric orbit as the research subject, as is shown in Figure 1, where *C* indicates the center of mass of an object, *h* represents the sensitive cavity, which is fixed to the satellite, then the position vector $r_{h}$ from the center of the sensitive cavity to the center of mass (CoM) of the satellite is constant, and the position vector of test mass relative to the satellite is $r\;=\;r_{h}\;+\;r_{rel}$. The relative translation equations of motion in the inertial system are first transformed into the satellite body coordinate system, similarly the relative attitude equations of motion are projected into the TM body coordinate system, which is illustrated in Figure 2. Then a comprehensive drag-free satellite dynamics model can be established as follows.

(1) $${\ddot{\varphi }}_{\mathrm{sc}}=I_{\mathrm{sc}}^{-1}\left(T_{\mathrm{Csc}}+w_{T_{\mathrm{Csc}}}+T_{Dsc}\right),$$

(2) $$\begin{array}{cc} {\ddot{r}}_{rel}^{h} & =\frac{1}{m_{tm}}\left(F_{Gtm}^{h}+F_{Dtm}^{h}+F_{SCtm}^{h}\right)-\frac{1}{m_{sc}}\left(F_{Gsc}^{h}+F_{Csc}^{h}+F_{Dsc}^{h}+F_{TMsc}^{h}\right)\\ \; & -2\omega_{sc}^{h}\times {\dot{r}}_{rel}^{h}-\omega_{sc}^{h}\times \left(\omega_{sc}^{h}\times \left(r_{h}^{h}+r_{rel}^{h}\right)\right)-{\dot{\omega }}_{sc}^{h}\times \left(r_{h}^{h}+r_{rel}^{h}\right), \end{array}$$

(3) $$\begin{array}{cc} {\ddot{\varphi }}_{rel}^{tm} & ={\left(I_{tm}\right)}^{-1}\left[-\left(\omega_{rel}^{tm}\omega_{sc}^{tm}\right)\times \left(I_{tm}\left(\omega_{rel}^{tm}+\omega_{sc}^{tm}\right)\right)\right]+{\left(I_{tm}\right)}^{-1}\left[T_{Gtm}^{tm}+T_{Dtm}^{tm}+T_{SCtm}^{tm}\right]\\ \; & -A_{TS}{\dot{\omega }}_{sc}^{sc}-A_{TS}\omega_{sc}^{sc}\times \omega_{rel}^{tm} \end{array}$$

where $w_{T_{\mathrm{Csc}}}$ indicates input noise, here assume that all input forces and moments acting on the satellite, as well as the measurement output of the sensor, are subject to noise, $T_{Dsc}$ denotes the disturbance moment to the satellite, $F_{Dtm}$ and $F_{Dsc}$ denote the test mass and the disturbance force on the satellite, respectively, and $T_{Dtm}$ indicates the disturbance moment to the test mass. The sc, tm, rel, *C*, *D*, and *G* subscripts indicate the S/C, Test Mass, measurements RELated to the sensitive cavity, Control command, Disturbance, and Gravity. The *h* superscript indicates the components in the sensitive cavity frame, and $A_{TS}$ is the coordinate transformation from the satellite frame to the test mass frame.

The dynamic model is expressed in the form of state space equations which are presented as follows:

(4) $$\begin{array}{cc} {\dot{X}}_{0} & =A_{0}X_{0}+B_{0}\left(u+w+f\right)\\ Y & =C_{0}X_{0}+d \end{array}$$

where

$$\begin{array}{c} A_{0}=\left[\begin{array}{cccccc} 0_{3} & I_{3} & 0_{3} & 0_{3} & 0_{3} & 0_{3}\\ 0_{3} & 0_{3} & 0_{3} & 0_{3} & 0_{3} & 0_{3}\\ 0_{3} & 0_{3} & 0_{3} & I_{3} & 0_{3} & 0_{3}\\ 0_{3} & 0_{3} & -\frac{K_{trans}}{m_{tm}} & -\frac{D_{trans}}{m_{tm}} & 0_{3} & 0_{3}\\ 0_{3} & 0_{3} & 0_{3} & 0_{3} & 0_{3} & 0_{3}\\ 0_{3} & 0_{3} & 0_{3} & 0_{3} & I_{tm}^{-1}K_{rot} & I_{tm}^{-1}D_{rot} \end{array}\right],B_{0}=\left[\begin{array}{ccc} 0_{3} & 0_{3} & 0_{3}\\ I_{sc}^{-1} & 0_{3} & 0_{3}\\ 0_{3} & 0_{3} & 0_{3}\\ 0_{3} & -\frac{I_{3}}{m_{sc}} & 0_{3}\\ 0_{3} & 0_{3} & 0_{3}\\ -I_{sc}^{-1} & 0_{3} & I_{tm}^{-1} \end{array}\right],\\ C_{0}=\left[\begin{array}{cccccc} I_{3} & 0_{3} & 0_{3} & 0_{3} & 0_{3} & 0_{3}\\ 0_{3} & 0_{3} & I_{3} & 0_{3} & 0_{3} & 0_{3}\\ 0_{3} & 0_{3} & 0_{3} & 0_{3} & I_{3} & 0_{3} \end{array}\right] \end{array}$$

where $K_{trans}$, $D_{trans}$, $K_{rot}$, $D_{rot}$ are the coupling coefficient matrices for translation and rotation, respectively. *u* is the system control variable, *w* is input noise, *d* is measurement noise, and *f* represents the total perturbation affecting the system, including the known part and the unmodeled part.

The Gravitational Wave Detector-TianQin requires detection satellites in deep-space orbit. The main disturbance on the satellite comes from solar pressure. To ensure a steady power supply and minimize fluctuations in the satellite’s internal thermal environment for ultra-stability, the drag-free satellite uses a body-attached battery array.

**Figure 3:**
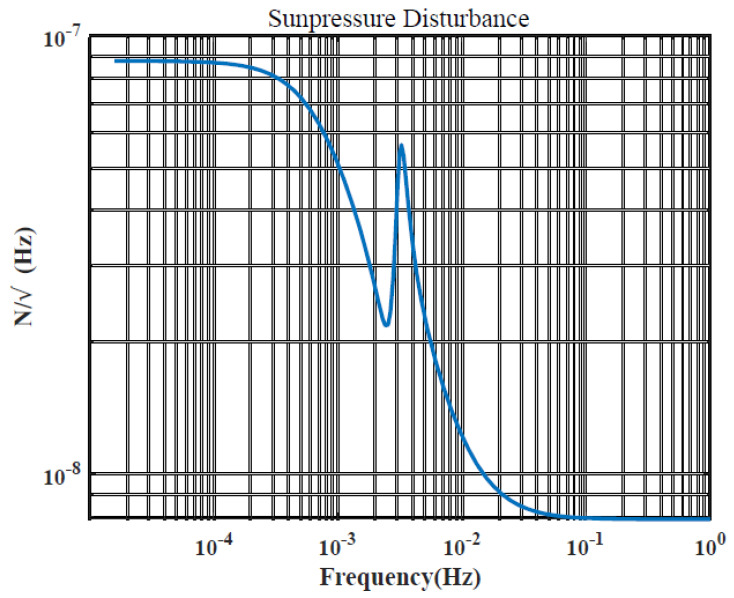
Solar pressure amplitude spectrum density.

The expression of this perturbation is shown below:

(5) $${\vec{F}}_{r}=-kC_{R}\rho_{SR}\left(\frac{S_{R}}{m}\right){\vec{r}}_{s}$$

where $C_{R}$ indicates surface reflection coefficient, normally 1–1.44, $\rho_{SR}$ indicates the solar pressure near the Earth, $4.56\times 10^{-6}\;N/m^{2}$, $\left(\frac{S_{R}}{m}\right)$ is the surface-to-mass ratio of the spacecraft, $S_{R}$ is the projected area of the spacecraft facing the sun, ${\vec{r}}_{s}$ is the unit vector indicating the direction from the center of the Earth to the sun. The sun exposure factor, denoted as *k*, is assumed to be 1 for the light area and 0 for the ground shadow area. The amplitude spectral density of the solar pressure on the satellite is shown in Figure 3.

**Figure 4:**
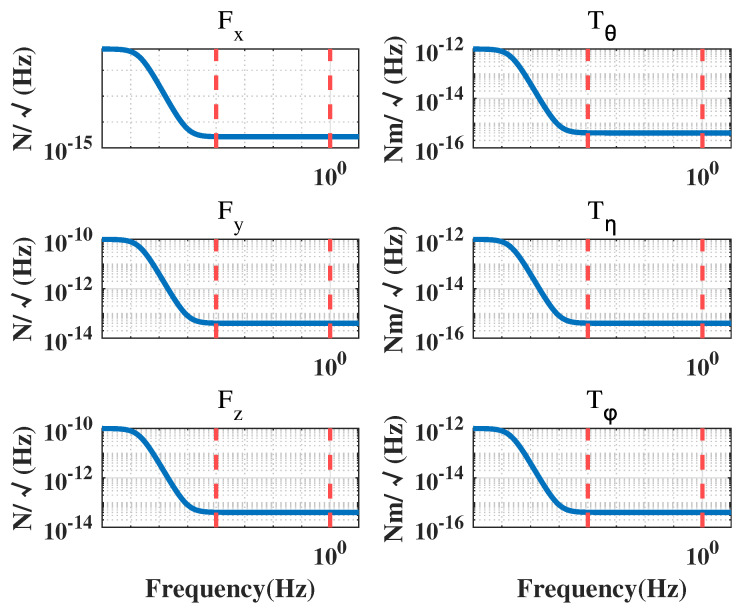
Electrostatic suspension actuation noise.

**Figure 5:**
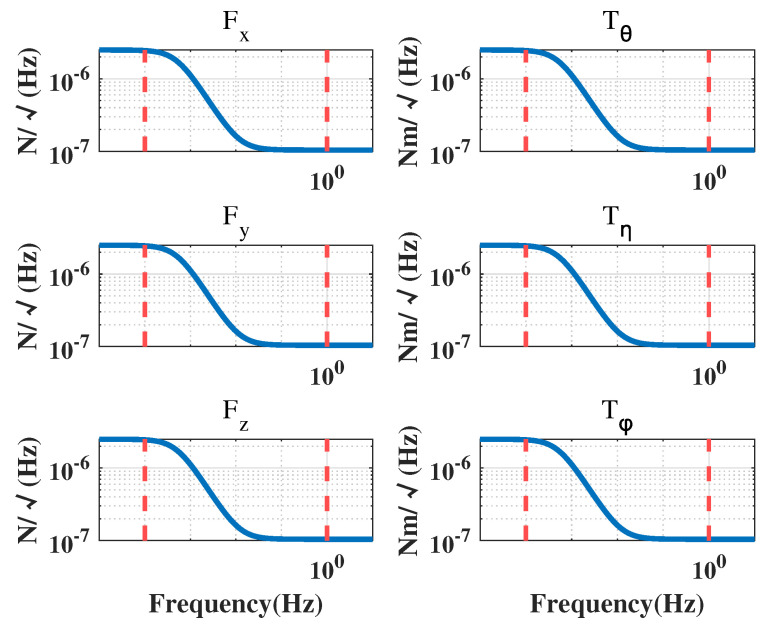
Thruster force noise.

**Figure 6:**
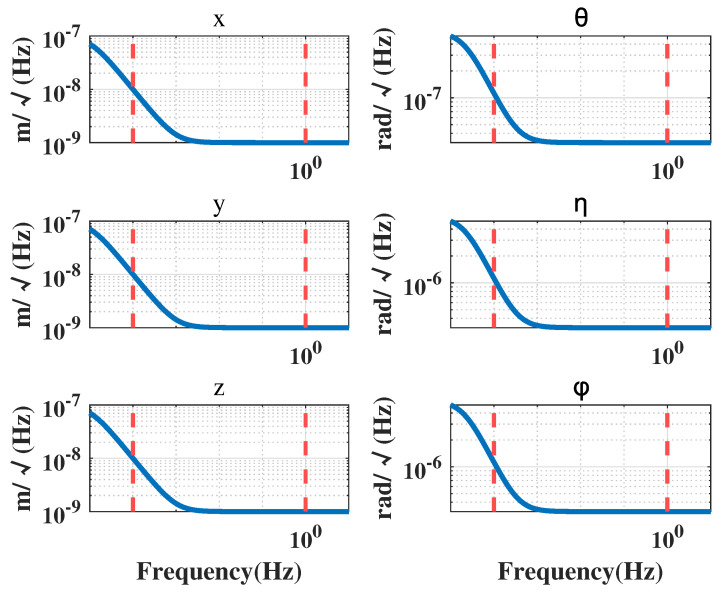
Inertial sensor measurement noise.

In a drag-free satellite with a single test mass, the displacement measurements between the CoM of the TM and the CoM of the satellite, as well as the attitude measurements of the TM relative to the satellite, are obtained by an inertial sensor. The attitude of the satellite is determined through a star sensor, and the micro-propulsion provides the necessary control forces and moments to maintain the desired position and attitude of the satellite. At present, the typical micro-propulsion systems have a noise power spectral density of $1\times 10^{-6}\;N/\sqrt{\mathrm{Hz}}$ under open-loop condition [22]. Their corresponding power spectral densities are presented in Figure 4, Figure 5 and Figure 6.

Based on the data of Ref. [23], it can be inferred that electrostatic actuation noise can be treated as white noise in the frequency band needed for the detection of gravitational waves by the TianQin detector.

In the case of capacitive displacement sensors, the noise levels for displacement measurements are equal in the x, y, and z directions, while the noise levels for angle measurements in the θ direction are one order of magnitude lower than those in the η and ϕ directions [23]. Figure 6 displays the measurement noise in each direction.

The spectral density curves of perturbation and noise are given in Figure 3, Figure 4, Figure 5 and Figure 6, which will be used as the basis for the modeling and simulation calculations later.

## 3. Extended State Kalman Filter Design

Achieving high accuracy of relative displacement and relative attitude control in a noisy and disturbed environment requires multiple steps, including disturbance estimation, noise suppression, and state control.

The Extended State Kalman Filter (ESKF) can estimate nonlinear uncertainty. In cases of initial error, uncertain dynamics (perturbation), and bounded noise, the perturbation is estimated and compensated for by the extended state, and noise effects can be suppressed. This paper uses ESKF to estimate disturbance forces and moments, such as solar pressure on the satellite and anomalous electromagnetic forces and moments on the test mass. First, we present the design scheme of ESKF, then apply it to uncertain disturbance estimation in drag-free control.

### 3.1. Extended State Kalman Filter

For the following discrete system containing uncertain perturbations

(6) $$\left\{\begin{array}{c} W_{k+1}=A_{k}W_{k}+B_{k}f\left(W_{k},k\right)+w_{k}\\ Y_{k}=C_{k}W_{k}+n_{k} \end{array}\right.,k=0,1,2,\ldots ,$$

where $W_{k}$ is the system state, $A_{k},B_{k}$ are system matrices, $C_{k}$ is the measurement matrix, $f\left(W_{k},k\right)$ is the nonlinear uncertain part in the system (6), $w_{k},n_{k}$ are the process noise and measurement noise, respectively, and $Y_{k}$ is the system measurement output. Treating $f\left(W_{k},k\right)$ as an additional state variable $f_{k}$, which is then estimated and compensated for. The extended system is described as

(7) $$\left\{\begin{array}{c} \left[\begin{array}{c} W_{k+1}\\ f_{k+1} \end{array}\right]={A_{k}}^{\prime }\left[\begin{array}{c} W_{k}\\ f_{k} \end{array}\right]+{B_{k}}^{\prime }G_{k}+\left[\begin{array}{c} w_{k}\\ 0 \end{array}\right]\\ Y_{k}={C_{k}}^{\prime }\left[\begin{array}{c} W_{k}\\ f_{k} \end{array}\right]+n_{k} \end{array}\right.$$

where $A_{k}^{\prime }=\left[\begin{array}{cc} A_{k} & B_{k}\\ 0 & I \end{array}\right]$, $B_{k}^{\prime }=\left[\begin{array}{c} 0\\ I \end{array}\right]$, $C_{k}^{\prime }=\left[\begin{array}{cc} C_{k} & 0 \end{array}\right]$, $f_{k}=f\left(W_{k},k\right)$, $G_{k}\overset{\Delta }{=}f_{k+1}-f_{k}$, assume $w_{k}$, $n_{k}$ are unrelated zero-mean Gaussian random series and $E\left(n_{k}{n_{k}}^{T}\right)\leq R_{k}$, $E\left(w_{k}{w_{k}}^{T}\right)\leq S_{k}$, $E\left(\left[\begin{array}{c} W_{0}-{\hat{W}}_{0}\\ f_{0}-{\hat{f}}_{0} \end{array}\right]{\left[\begin{array}{c} W_{0}-{\hat{W}}_{0}\\ f_{0}-{\hat{f}}_{0} \end{array}\right]}^{T}\right)\leq P_{0}$, ${\hat{W}}_{0}$ is the estimation of $W_{0}$, ${\hat{f}}_{0}$ is the initial value of the nominal part of $f(W_{k},k)$, $P_{0}$ is a known constant matrix, and $E\left(G_{i}^{2}\right)\leq {\bar{q}}_{i},i=1,2,\ldots$, $q_{i}$ is bounded.

According to the classical state observer theory, the extended state observer for the extended state Equation (7) is shown below, where $X_{k+1}=\left[\begin{array}{c} W_{k+1}\\ f_{k+1} \end{array}\right]$

(8) $${\hat{X}}_{k+1}=A_{k}{\hat{X}}_{k}+B_{k}{\hat{G}}_{k}-K_{k}\left(Y_{k}-C_{k}{\hat{X}}_{k}\right)$$

Based on the given initial estimation ${\hat{X}}_{0}$ and initial value of covariance matrix $P_{0}$, we obtain the middle value of the estimated quantity ${\hat{X}}_{k}^{-}$ and update the value of the covariance matrix ${P_{k}}^{-}$

(9) $${\hat{X}}_{k}^{-}=A_{k}^{\prime }{\hat{X}}_{k-1}+B_{k}^{\prime }u_{k-1}+B_{e}{\hat{G}}_{k}$$

(10) $$\begin{array}{cc} {P_{k}}^{-} & =\left(1+\theta \right)A_{k}^{\prime }P_{k-1}{A_{k}^{\prime }}^{T}+\left(1+\frac{1}{\theta }\right)Q_{1,k-1}+Q_{2,k-1} \end{array}$$

where $\theta =\sqrt{\frac{\mathrm{tr}\left(Q_{1,0}\right)}{\mathrm{tr}\left(P_{0}\right)}}$ is used to decouple the cross terms of estimation error and uncertainty, $Q_{1,0}=4B_{e}Q_{0}{B_{e}}^{T}$, $Q_{2,0}=B_{k}^{\prime }S_{k}{B_{k}^{\prime }}^{T}$, $S_{k}$ is the variance of $w_{k}$, $R_{k}$ is the variance of $n_{k}$, ${\hat{G}}_{k}$ is the estimation of $G_{k}$, whose value is calculated by ${\hat{G}}_{k}=sat\left({\bar{G}}_{k},\sqrt{q_{i}}\right)$, where

(11) $$sat\left(f,b\right)=\{\begin{array}{cc} \;b & f>b\\ \begin{array}{c} \;f\\ \;-b \end{array} & \begin{array}{c} b>f>-b\\ -b>f \end{array} \end{array}$$

by calculating $K_{k}$, update the estimation ${\hat{X}}_{k}$ and the covariance matrix $P_{k}$

(12) $$K_{k}={P_{k}}^{-}{C_{k}^{\prime }}^{T}{\left({C^{\prime }}_{k}{P_{k}}^{-}{{C^{\prime }}_{k}}^{T}+R_{k}\right)}^{-1}$$

(13) $${\hat{X}}_{k}={\hat{X}}_{k}^{-}+K_{k}\left(Y_{k}-{C^{\prime }}_{k}{\hat{X}}_{k}^{-}\right)$$

(14) $$P_{k}=\left(I-K_{k}C_{k}^{\prime }\right){P_{k}}^{-}{\left(I-K_{k}C_{k}^{\prime }\right)}^{T}+K_{k}R_{k}{K_{k}}^{T}$$

**Figure 7:**
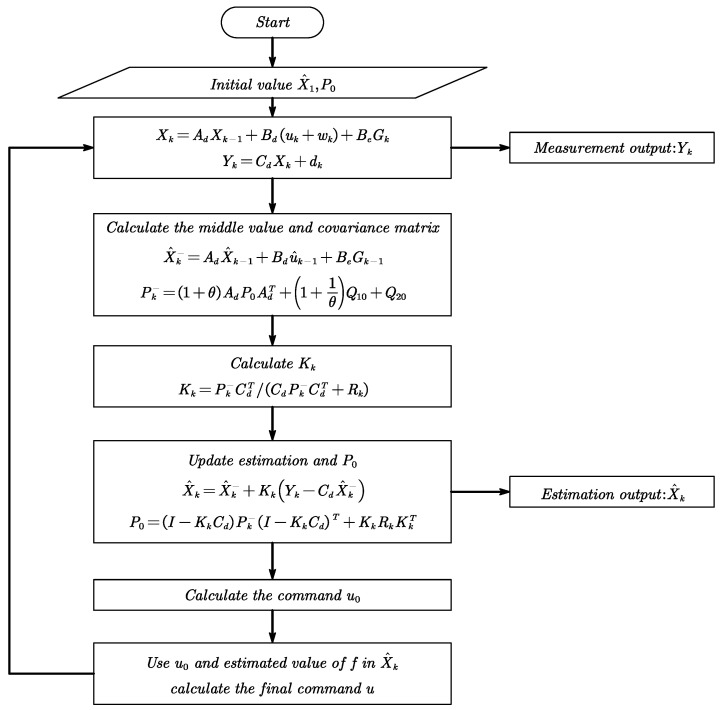
ESKF calculation flow chart.

After that, calculate the control variable $u_{0}$ based on the error between the state estimate and the reference, then the calculated control variable $u_{0}$ and the estimated value $\hat{f}$ of the disturbance are used to calculate the final control variable *u*, and the flow chart is shown in Figure 7.

### 3.2. Extended State Design of Drag-Free Control System

The complex space environment presents a challenge in accurately modeling and describing perturbations affecting on satellite and test masses. Conventional control methods are model-dependent, and their performance can be severely affected by the inaccuracies of the perturbation model. To address this issue, Section 3.1 proposes a method to expand the perturbations into new states and create a new filter model. Then the perturbation can be compensated and the noise can be suppressed.

The uncertain disturbance term is $f={\left[T_{Dsc},F_{Dsc},T_{Dtm}\right]}^{T}$. $T_{Dsc}$ represents the disturbance moments that the satellite is subjected to, $F_{Dsc}$ represents the disturbance forces that the satellite is subjected to, and $T_{Dtm}$ represents the disturbance moment that TM is subjected to. Those are treated as extended states. The state vector is taken as $X\;=\;{\left[\varphi_{sc},{\dot{\varphi }}_{sc},r_{rel},{\dot{r}}_{rel},\varphi_{rel},{\dot{\varphi }}_{rel},T_{Dsc},F_{Dsc},T_{Dtm}\right]}^{T}$, $Y={\left[\varphi_{sc},r_{rel},\varphi_{rel}\right]}^{T}$ indicates related attitude and displacement that can be measured.

According to Section 3.1, the extended state differential equation is obtained as

(15) $$\begin{array}{c} \dot{X}=AX+B\left(u+w\right)+B_{e}\dot{f}\\ Y=CX+d \end{array}$$

where $A=\left[\begin{array}{cc} A_{0} & B_{0}\\ 0 & I \end{array}\right],B=\left[\begin{array}{c} B_{0}\\ 0 \end{array}\right],C=\left[\begin{array}{cc} C_{0} & 0 \end{array}\right]$, $B_{e}={\left[0,0,1\right]}^{T}$, symbols has the same physical meaning as in Equation (4).

The extended state Equation (15) is discretized to obtain the discrete difference equation model (16)

(16) $$\begin{array}{cc} X_{k+1} & =A_{d}X_{k}+B_{d}\left(u+w\right)+B_{d}G_{k}\\ Y_{k} & =C_{d}X_{k}+d_{k} \end{array}$$

where $G_{k}\overset{\Delta }{=}f_{k+1}-f_{k}$.

The ESKF is designed according to the flowchart shown in Figure 7 to estimate the kinematic state parameters and the unknown disturbances, while implementing feedback control. Where $q_{i}={\left(\max\left|f_{i+1}-f_{i}\right|\right)}^{2}$, $Q_{0}=i\mathrm{diag}\left(q_{i}\right)$, *i* is the number of state vector.

## 4. Feedback Controller Design

Once the estimation of states and perturbations has been successfully obtained, the next step is the selection of an appropriate controller. The Linear Quadratic Regulator (LQR) is a control strategy designed to minimize a cost function. The optimal control law is obtained through the design of the state feedback controller, which allows for the completion of closed-loop optimal control in a fast, stable, and accurate manner.

The performance index for LQR control reflects the requirements for both state and control quantities, and the cost function used in this paper is

(17) $$J=\sum_{0}^{n}\left(x{\left(k\right)}^{T}Qx\left(k\right)+u{\left(k\right)}^{T}Ru\left(k\right)\right)$$

The weighting matrix *Q* is semi-positive definite and *R* is positive definite, which are set as a diagonal matrix in the subsequent simulation. For the first term in the cost function *J*, each component is required to be small in the control process. The larger the weight in *Q* means the stricter the constraint on the components; while the second term in the cost function indicates the requirement for the control output, which is weighted according to the different characteristics of each component.

The Ricatti equation $PA+A^{T}P-PBR^{-1}B^{T}P+Q=0$ is used to calculate *P*, then based on $K=R^{-1}B^{T}P$, the feedback gain matrix is calculated, the control law of LQR is chosen as $u\left(k\right)=-K\hat{x}\left(k\right)$.

In order to maximize the effective time of gravitational wave detection and minimize the output of thrusters (actuators), it is necessary to ensure that the detection satellite is maintained in a free and stable flight for as long a time as possible. To achieve this goal, the adjustment transition process of drag-free control needs to be as fast as possible, allowing the detection system to quickly reach an ultra-static and ultra-stable state. Our research shows that the LQR control strategy can satisfy the optimal control requirements, enabling a rapid transition to a steady state and achieving the required control accuracy. By appropriately designing the weights of each component, in addition to the values of *Q* and *R*, it is possible to achieve a more refined control.

Nonlinear error feedback is used in classical Active Disturbance Rejection Control [1], mainly by rewriting the weighting of classical PID control into a nonlinear combination, as shown in Equation (18)

(18) $$fal\left(e,\alpha ,\delta \right)=\{\begin{array}{c} \;\frac{e}{\delta^{\alpha -1}},\left|e\right|\leq \delta \\ \;\mathrm{sgn}\left(e\right){\left|e\right|}^{\alpha },\left|e\right|>\delta \end{array}$$

It is a continuous power series function with linear segments near the origin, δ>0 denotes the length of the interval of the linear segment, and *e* indicates the amount of error.

This function fal() is characterized by increasing the gain when the error is small and using a small gain when the error is large, which prevents high-frequency chattering due to excessive gain calculated when the error is small [24,25]. The control law for nonlinear error feedback is

(19) $$\begin{array}{cc} u & =\gamma_{1}fal\left(e_{1},\alpha_{1},\delta \right)+\gamma_{2}fal\left(e_{2},\alpha_{2},\delta \right)+\gamma_{3}fal\left(e_{3},\alpha_{3},\delta \right) \end{array}$$

where $e_{1}\left(k\right)=r-\hat{x}\left(k\right),e_{2}\left(k\right)=\sum_{0}^{k}\left(r-\hat{x}\left(k\right)\right),e_{3}=\dot{r}-\hat{\dot{x}}\left(k\right)$. Parameter triples $\gamma_{1},\gamma_{2},\gamma_{3}$ determine the final control variable, the value of δ is generally selected as the sampling time for discrete systems, the value of α satisfies $\alpha \in \left(0,1\right)$.

A simulation program using the combination of ESKF and LQR is developed, then the comparison results of the combination of ESKF+NSEF and the combination of ESKF+LQR is presented and analyzed in the next section.

## 5. Simulation Analysis

**Figure 8:**
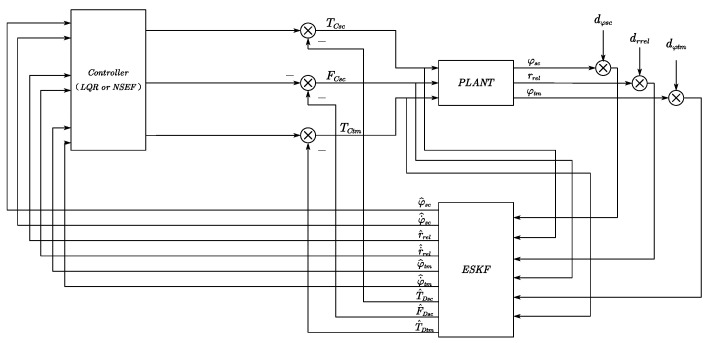
ESKF-based ADRC system design.

According to the discrete model (16), a block diagram of the drag-free satellite control system is designed and presented in Figure 8. The measurement mechanism provides information on the attitude angle of the satellite, the displacement, and the attitude of the test mass relative to the center of the cavity. The output command of actuators and output measurement information of the sensors are both inputs of ESKF. The ESKF estimates the attitude, angular velocity, displacement, velocity, disturbance forces, and disturbance moments. By selecting an appropriate controller with the ESKF, high-accuracy anti-disturbance control of the drag-free satellite/test mass dynamic system is achieved.

First, we consider the LQR controller. The ESKF filter design discussed in Section 3 was simulated in MATLAB with a time step of 0.01 s. The ESKF consists of 27 states, which are divided into three groups of nine states each. After the ESKF output was stable and tracking accurately. The LQR control algorithm was then employed for designing the control law, and the corresponding simulation results were obtained.

Initial conditions of simulation:

The perturbation forces and moments of the satellite are modeled as constant superposition sinusoidal perturbations with phase differences in each axial direction. Specifically: $F_{Dsc}\;=\;\left[\begin{array}{c} -12.8+7.7\times \sin(\omega_{d}t)\\ -12.8+7.7\times \sin(\omega_{d}t+\frac{2\pi }{3})\\ -12.8+7.7\times \sin(\omega_{d}t+\frac{4\pi }{3}) \end{array}\right]\;\times \;10^{-7}\left(N\right)$, the disturbance moment to the satellite is modeled as $T_{Dsc}\;=\;\left[\begin{array}{c} -1.2+6.6\times \sin\left(\omega_{d}t\right)\\ -1.2+6.6\times \sin\left(\omega_{d}t+\frac{2\pi }{3}\right)\\ -1.2+6.6\times \sin\left(\omega_{d}t+\frac{4\pi }{3}\right) \end{array}\right]\;\times \;10^{-6}\left(N\cdot m\right)$, moment of disturbance to the test mass is $T_{D\mathrm{tm}}\;=\;\left[\begin{array}{c} -1.2+7.7\times \sin\left(\omega_{d}t\right)\\ -1.2+7.7\times \sin\left(\omega_{d}t+\frac{2\pi }{3}\right)\\ -1.2+7.7\times \sin\left(\omega_{d}t+\frac{4\pi }{3}\right) \end{array}\right]\;\times \;10^{-12}\left(N\cdot m\right)$, where $\omega_{d}=1.2\times 10^{-3}\;\mathrm{Hz}$. The simulation program will achieve real-time estimation of the extended state for the above perturbations, and the results of the error analysis of the estimation are given later. The expectation of the input noise of the thrusters providing force and moment to the satellite are $1\times 10^{-9}\;N/\sqrt{\mathrm{Hz}}$ and $1\times 10^{-9}\;N\cdot m/\sqrt{\mathrm{Hz}}$, respectively. The input noise expectation of the electrostatic actuator providing the test mass control torque is $1\times 10^{-15}\;N\cdot m/\sqrt{\mathrm{Hz}}$, the expectation of the measurement noise of the star sensor providing satellite attitude measurement is set as $1\times 10^{-7}\;\mathrm{rad}/\sqrt{\mathrm{Hz}}$. And the expectation of displacement measurement noise of inertial sensor is $1\times 10^{-9}\;m/\sqrt{\mathrm{Hz}}$, the expectation of attitude measurement noise is $1\times 10^{-8}\;\mathrm{rad}/\sqrt{\mathrm{Hz}}$. To meet the requirements of gravitational wave detection, the control loop’s design objective is set as follows: the setting time should be less than 1 min, and the amplitude spectral density of the relative displacement between test mass and satellite should both be less than $10^{-8}\;m/\sqrt{\mathrm{Hz}}$ within the detection frequency band.

The performance of the combination of LQR and ESKF is evaluated initially. Subsequently, the performance of using the combination of LQR and ESKF is compared with the performance of the combination of NSEF and ESKF in terms of control accuracy and setting time. The intrinsic reasons for any differences observed are analyzed, and recommendations for engineering design are provided.

### 5.1. ESFK+LQR

By utilizing the ESKF-estimated states as input, we designed an LQR controller based on Equation (17). The initial value of state estimation was ${\hat{X}}_{0}={\left[0,0,0,0,0,0,0,0,0\right]}^{T}$, and the values of *Q* and *R* were selected as $R=1\times 10^{-4}\mathrm{diag}\left(I_{3},10I_{3},I_{3}\right)$. It should be noted that a larger value of *Q* can facilitate faster convergence of the states for the LQR controller.

#### 5.1.1. Satellite Attitude

**Figure 9:**
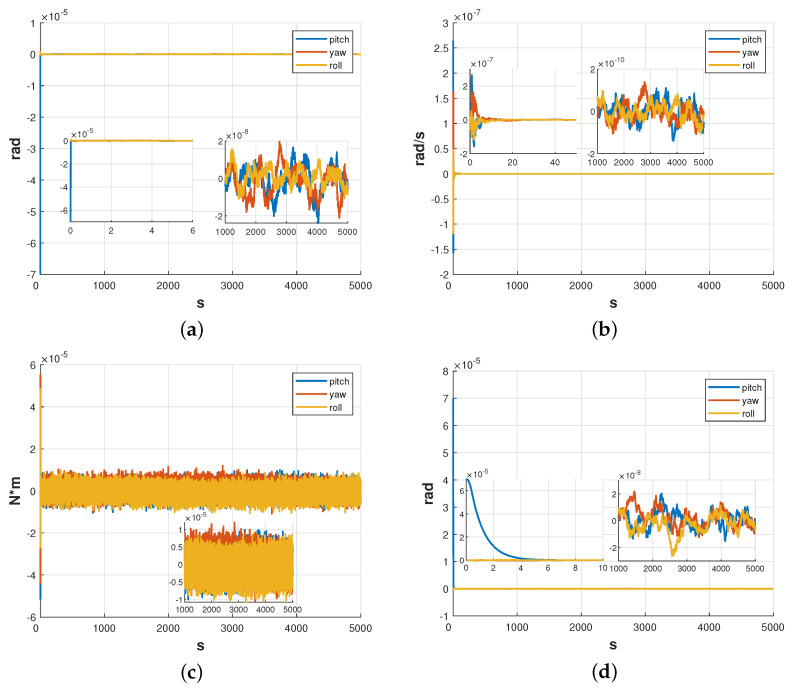
Simulation results of satellite attitude. (**a**) Estimation error of the attitude angle. (**b**) Estimation error of the attitude angular velocity. (**c**) Estimation error of disturbance moment. (**d**) Attitude control results.

Figure 9 demonstrates the effectiveness of the ESKF and LQR controllers in controlling the attitude angle of the satellite. The results show that the attitude angle was successfully controlled from the initial $\varphi_{sc}={\left[7\times 10^{-5},0,0\right]}^{T}\;\mathrm{rad}$ to $\pm 3\times 10^{-8}\;\mathrm{rad}$ for all three attitude angles with a setting time of about 7 s, achieving the desired control target. The estimation error of the attitude angle was measured to be $\pm 2\times 10^{-8}\;\mathrm{rad}$, while the estimation error of the attitude angular velocity and disturbance moment were $\pm 2\times 10^{-10}\;\mathrm{rad}/s$ and $\pm 1\times 10^{-5}\;N\cdot m$, respectively.

**Figure 10:**
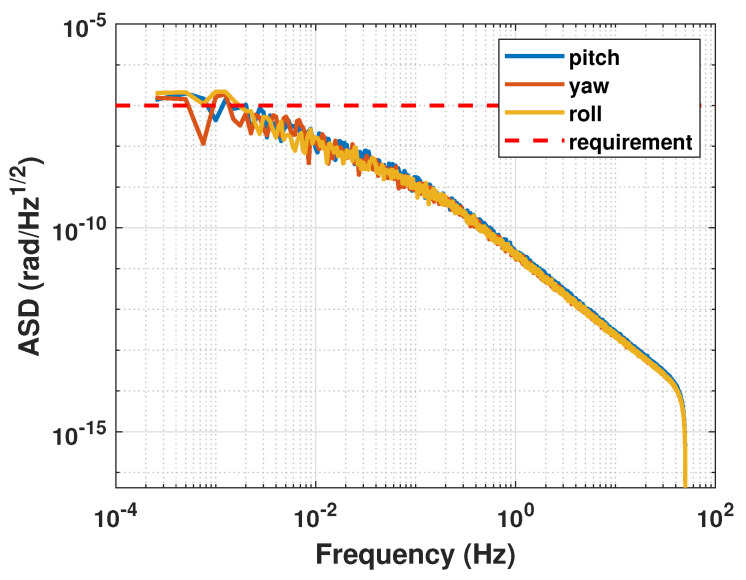
Amplitude spectral density of attitude of the satellite.

The amplitude spectral density curves for each attitude angle control error are presented in Figure 10. Based on the results shown in Figure 10, it can be observed that within the measurement bandwidth, the amplitude spectral density of the satellite’s attitude control error conforms to the design requirements of $10^{-7}\;\mathrm{rad}/\sqrt{\mathrm{Hz}}$.

#### 5.1.2. Test Mass and Satellite Relative Displacement

**Figure 11:**
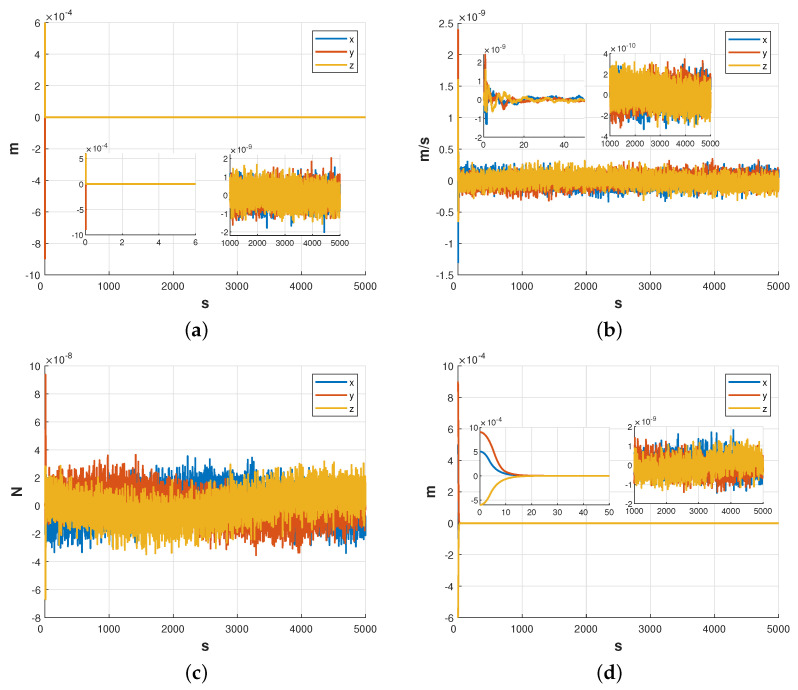
Simulation results of relative displacement between test mass and satellite. (**a**) Estimation error of the relative displacement. (**b**) Estimation error of the velocity. (**c**) Estimation error of disturbance. (**d**) Control results of relative displacement.

The results in Figure 11 show that the relative displacement was successfully controlled from $r_{rel}={\left[0.0005,0.0009,-0.0006\right]}^{T}\;m$ to $\pm 2\times 10^{-9}\;m$ for all three axes within an adjustment time of 20 s, achieving the desired control target. The position estimation error was measured to be $\pm 2.2\times 10^{-9}\;m$, while the velocity estimation error and disturbance estimation error were $\pm 4\times 10^{-10}\;m/s$ and $\pm 4\times 10^{-8}\;N$, respectively.

**Figure 12:**
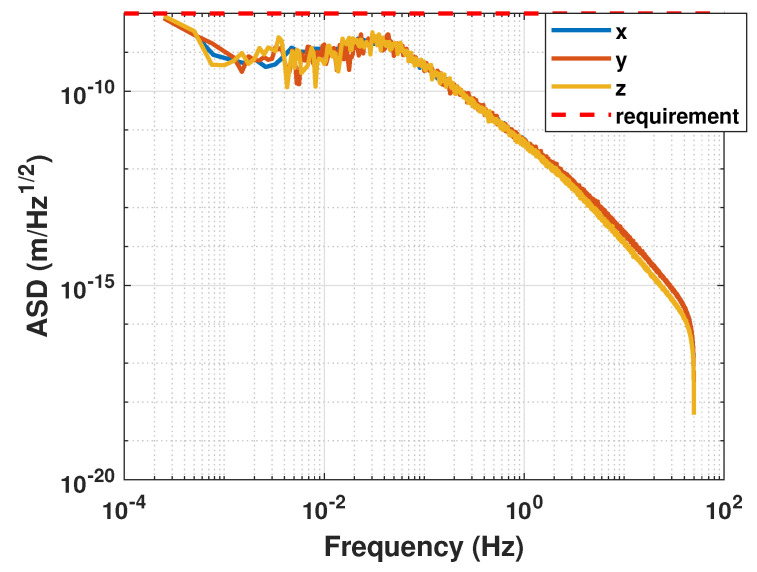
Amplitude spectral density of relative displacement.

The amplitude spectral density curves for each axial direction are presented in Figure 12. Based on the observations from Figure 12, it can be concluded that the kinematic indexes of the translation within the frequency band satisfy the design requirements and achieve $10^{-8}\;m/\sqrt{\mathrm{Hz}}$.

#### 5.1.3. Relative Attitude between Test Mass and Satellite

**Figure 13:**
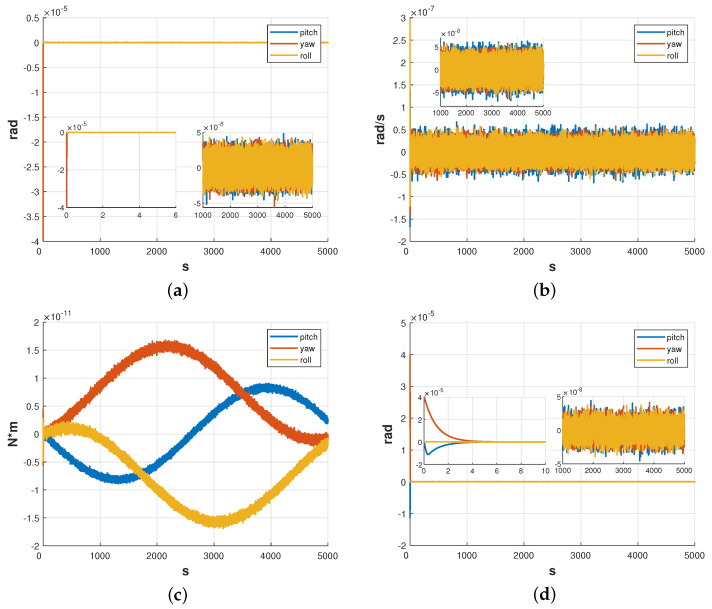
Simulation results of relative attitude between test mass and satellite. (**a**) Estimation error of the relative attitude. (**b**) Estimation error of the attitude angular velocity. (**c**) Estimation error of disturbance moment. (**d**) Relative attitude control results.

Figure 13 shows the simulation results of ESKF and LQR controller dealing with the relative attitude between test mass and satellite. Based on the results from Figure 13, it can be concluded that the ESKF and LQR controllers designed in this paper were capable of controlling the relative attitude between the test mass and the satellite from $\varphi_{rel}={\left[0,4\times 10^{-5},0\right]}^{T}\;\mathrm{rad}$ to $\pm 5\times 10^{-8}\;\mathrm{rad}$ for all three axes. The control result was found to be essentially oscillation-free, with an adjustment time of about 6s, achieving the desired control target. The estimation error of the attitude angle was measured to be $\pm 5\times 10^{-8}\;\mathrm{rad}$, while the estimation errors of the attitude angular velocity and disturbance moment were $\pm 6\times 10^{-8}\;\mathrm{rad}/s$ and $\pm 2\times 10^{-11}\;N\cdot m$, respectively.

**Figure 14:**
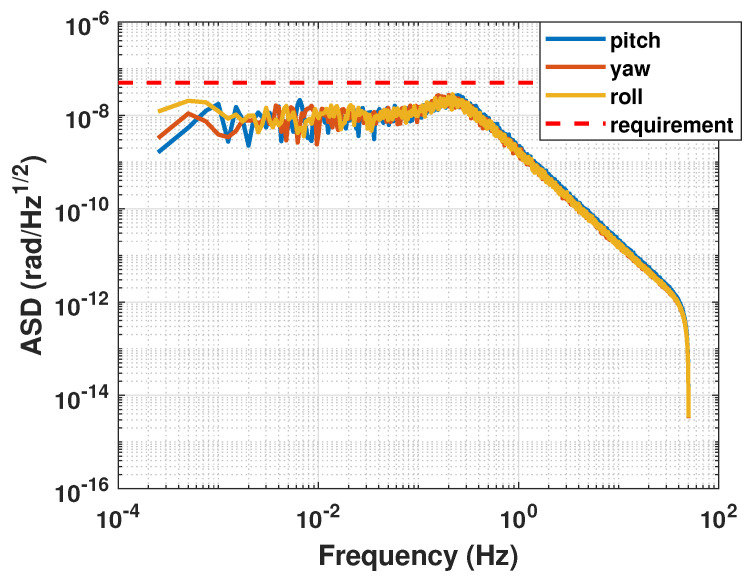
Amplitude Spectral Density of the related attitude between test mass and satellite.

The amplitude spectral density curves for each attitude angle are presented in Figure 14. The amplitude spectral densities are lower than $5\times 10^{-8}\;\mathrm{rad}/\sqrt{\mathrm{Hz}}$ in the whole frequency band.

### 5.2. Comparison Evaluation

**Figure 15:**
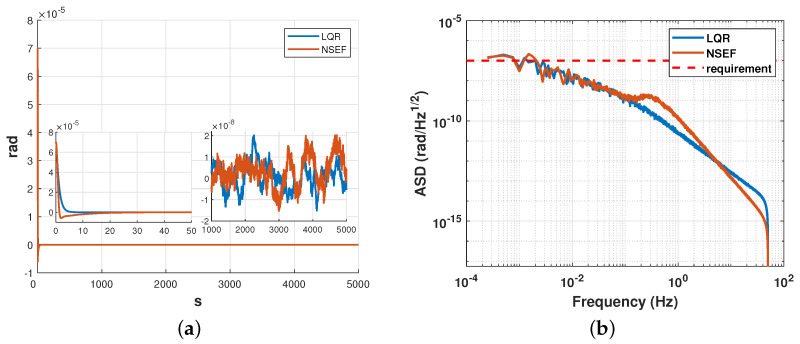
Simulation result of satellite attitude (pitch angle) between LQR and NSEF. (**a**) Control result in time domain. (**b**) Comparison of Amplitude Spectral Density.

**Figure 16:**
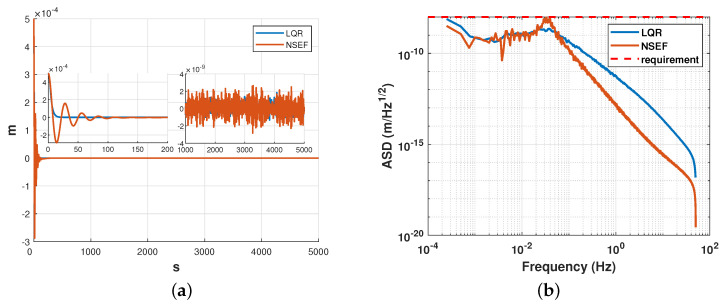
Simulation result of relative displacement (x-axis) between LQR and NSEF. (**a**) Control result in the time domain. (**b**) Comparison of Amplitude Spectral Density.

**Figure 17:**
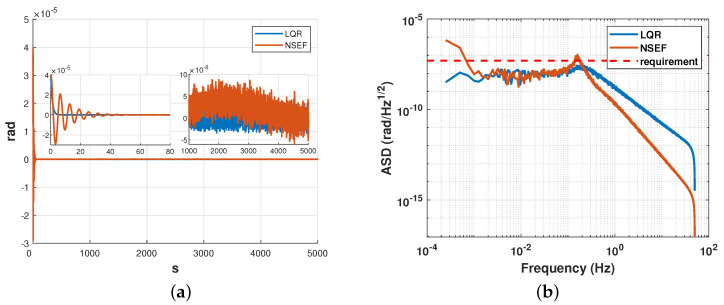
Simulation result of relative attitude (yaw angle) between LQR and NSEF. (**a**) Control result in time domain. (**b**) Comparison of Amplitude Spectral Density.

To demonstrate the efficacy of the designed method, we present a simulation to compare ESKF+LQR with ESKF+NSEF. This allows us to illustrate the functions of our objectives and evaluate their performance against an established method. Simulation results is given in Figure 15, Figure 16 and Figure 17

Since the convergence facility of the fal() function used in NSEF is mainly based on the value of α, while the proof of certain physical relation is extremely complicated, the value of α is determined by rule of thumb. Based on the method in Ref. [26], we take $\alpha_{1}=\alpha_{2}=\alpha_{3}=0.5$ in simulation. The simulation results of relative displacement of the x-axis are shown in Figure 16, while the control accuracy is satisfied, and the convergence process of ESKF+LQR was found to be much faster than that of ESKF+NSEF.

The RMS of the two approaches is listed in Table 1. Additionally, the LQR controller was observed to produce negligible oscillations in the system, which can be advantageous in terms of reducing energy consumption and extending the effective time of gravitational wave signal detection.

## 6. Conclusions

Integrating perturbations into states vector, the ESKF method demonstrates the capability to accurately estimate the state and disturbance of the drag-free satellite dynamics, and effectively suppressing handling noise. This approach lays the foundation for the controller to achieve accurate adjustment of the system state, and it is recommended to consider the ESKF method as a viable alternative for estimating uncertain disturbances in future drag-free engineering designs.

The LQR controller’s feedback parameter matrix can be rigorously derived using the generalized index to ensure optimal control performance. By combining the reasonable values of the state covariance matrix, the designed gain matrix guarantees the convergence speed of the system state, while the accurate estimation of ESKF enhances the relative attitude control accuracy by an order of magnitude.

For the relative displacement, the adjustment time of NSEF fails to reach the control task requirements. This is mainly due to the relatively slow control process of the relative kinematic parameters between the satellite and the test mass in NSEF, and the lack of a complete theoretical method to adjust and optimize the nonlinear error feedback parameters.

In summary, the ESKF+LQR control approach enables the system to reach a steady state rapidly and smoothly, thereby increasing the free flight time available for gravitational wave observation. This is more in line with the desired observation duration for gravitational wave detection. In comparison to the combination of ESKF+NSEF, the combination of ESKF+LQR is capable of adjusting to the reference target swiftly and reducing oscillations, leading to a reduction in thruster mass consumption.

## Figures and Tables

**Table 1:** RMS of two different control approaches.

Axis	ESKF+LQR	ESKF+NSEF
satellite attitude (pitch)	6.3076 × $10^{-9}$ rad	7.4918 × $10^{-9}$ rad
satellite attitude (yaw)	7.2104 × $10^{-9}$ rad	5.9713 × $10^{-9}$ rad
satellite attitude (roll)	7.4293 × $10^{-9}$ rad	4.7966 × $10^{-9}$ rad
relative displacement (x)	4.5918 × $10^{-10}$ m	8.9468 × $10^{-10}$ m
relative displacement (y)	4.5422 × $10^{-10}$ m	9.3517 × $10^{-10}$ m
relative displacement (z)	4.7442 × $10^{-10}$ m	9.4479 × $10^{-10}$ m
relative attitude (pitch)	1.0859 × $10^{-10}$ rad	2.7201 × $10^{-8}$ rad
relative attitude (yaw)	1.0480 × $10^{-10}$ rad	3.1758 × $10^{-8}$ rad
relative attitude (roll)	1.0299 × $10^{-10}$ rad	3.2608 × $10^{-8}$ rad

## Data Availability

Not applicable.

## Abbreviations

The following abbreviations are used in this manuscript:

ADRC	Active Disturbance Rejection Control
NSEF	Nonlinear State Error Feedback
LQR	Linear Quadratic Regulator
ESO	Extended State Observer
